# Outcomes with Finerenone in Participants with Stage 4 CKD and Type 2 Diabetes: A FIDELITY Subgroup Analysis

**DOI:** 10.2215/CJN.0000000000000149

**Published:** 2023-04-07

**Authors:** Pantelis Sarafidis, Rajiv Agarwal, Bertram Pitt, Christoph Wanner, Gerasimos Filippatos, John Boletis, Katherine R. Tuttle, Luis M. Ruilope, Peter Rossing, Robert Toto, Stefan D. Anker, Zhi-Hong Liu, Amer Joseph, Christiane Ahlers, Meike Brinker, Robert Lawatscheck, George Bakris

**Affiliations:** 1 Department of Nephrology, Hippokration Hospital, Aristotle University of Thessaloniki, Thessaloniki, Greece; 2 Richard L. Roudebush VA Medical Center and Indiana University, Indianapolis, Indiana; 3 Department of Medicine, University of Michigan School of Medicine, Ann Arbor, Michigan; 4 Medizinische Klinik und Poliklinik 1, Schwerpunkt Nephrologie, Universitätsklinik Würzburg, Germany; 5 National and Kapodistrian University of Athens, School of Medicine, Department of Cardiology, Attikon University Hospital, Athens, Greece; 6 Faculty of Medicine, Laiko General Hospital, University of Athens, Athens, Greece; 7 Providence Medical Research Center, Providence Health Care, Spokane, Washington; 8 Institute of Translational Health Sciences, Kidney Research Institute, and Nephrology Division, University of Washington, Seattle, Washington; 9 Cardiorenal Translational Laboratory and Hypertension Unit, Institute of Research imas12, Madrid, Spain; 10 CIBER-CV, Hospital Universitario 12 de Octubre, Madrid, Spain; 11 Faculty of Sport Sciences, European University of Madrid, Madrid, Spain; 12 Steno Diabetes Center Copenhagen, Herlev, Denmark; 13 Department of Clinical Medicine, University of Copenhagen, Copenhagen, Denmark; 14 Department of Internal Medicine, University of Texas Southwestern Medicine, Dallas, Texas; 15 Department of Cardiology (CVK) and Berlin Institute of Health Center for Regenerative Therapies, German Centre for Cardiovascular Research Partner Site Berlin, Charité Universitätsmedizin, Berlin, Germany; 16 National Clinical Research Center of Kidney Diseases, Jinling Hospital, Nanjing University School of Medicine, Nanjing, China; 17 Cardiology and Nephrology Clinical Development, Bayer AG, Berlin, Germany; 18 Statistics and Data Insights, Bayer AG, Wuppertal, Germany; 19 Cardiology and Nephrology Clinical Development, Bayer AG, Wuppertal, Germany; 20 Medical Affairs & Pharmacovigilance, Pharmaceuticals, Bayer AG, Berlin, Germany; 21 Department of Medicine, University of Chicago Medicine, Chicago, Illinois

**Keywords:** chronic kidney disease, diabetes mellitus

## Abstract

**Background:**

Patients with stage 4 CKD and type 2 diabetes have limited treatment options to reduce their persistent cardiovascular and kidney risk. In Finerenone in Chronic Kidney Disease and Type 2 Diabetes (FIDELITY), a prespecified pooled analysis of Finerenone in Reducing Kidney Failure and Disease Progression in Diabetic Kidney Disease (FIDELIO-DKD) and Finerenone in Reducing Cardiovascular Mortality and Morbidity in Diabetic Kidney Disease (FIGARO-DKD), finerenone improved heart-kidney outcomes in participants with CKD and type 2 diabetes.

**Methods:**

This FIDELITY subgroup analysis investigated the effects of finerenone in participants with stage 4 CKD (eGFR <30 ml/min per 1.73 m^2^). Efficacy outcomes included a cardiovascular composite (cardiovascular death, nonfatal myocardial infarction, nonfatal stroke, or hospitalization for heart failure) and a kidney composite (kidney failure, sustained ≥57% decrease in eGFR from baseline, or kidney disease death).

**Results:**

Of 13,023 participants, 890 (7%) had stage 4 CKD. The hazard ratio for risk of cardiovascular composite outcome with finerenone versus placebo was 0.78 (95% confidence interval, 0.57 to 1.07). The kidney composite outcome proportional hazards assumption was not met for the overall study period, with a protective effect only shown up to 2 years, after which the direction of association was inconsistent, and an observed loss of precision over time incurred on finerenone versus placebo risk differences. Nonetheless, albuminuria and rate of eGFR decline were consistently reduced with finerenone versus placebo. Adverse events were balanced between treatment arms. Hyperkalemia was the most common adverse event reported (stage 4 CKD: 26% and 13% for finerenone versus placebo, respectively); however, the incidence of hyperkalemia leading to permanent discontinuation was low (stage 4 CKD: 3% and 2% for finerenone versus placebo, respectively).

**Conclusions:**

The cardiovascular benefits and safety profile of finerenone in participants with stage 4 CKD were consistent with the overall FIDELITY population; this was also the case for albuminuria and the rate of eGFR decline. The effects on the composite kidney outcome were not consistent over time.

## Visual Abstract

**Figure:**
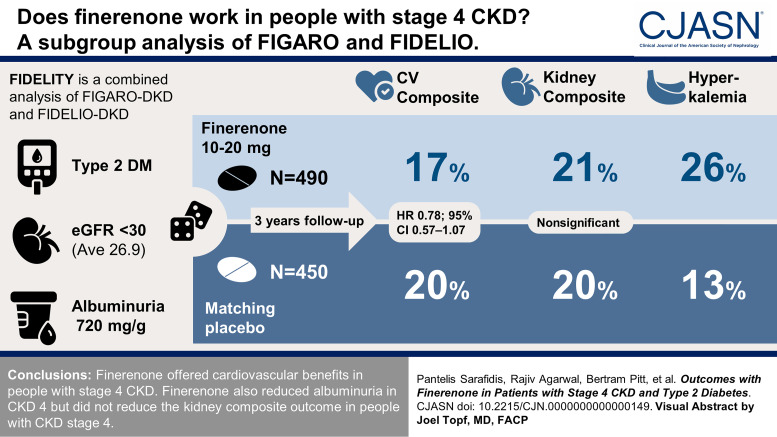

## Introduction

Despite recent advances in CKD and type 2 diabetes mellitus management, treatment options remain limited for patients with stage 4 CKD (eGFR <30 ml/min per 1.73 m^2^).^1,2^ In addition, in patients with stage 4 CKD, cardiovascular mortality is three times higher than in those with normal kidney function.^3^ Sodium-glucose cotransporter-2 inhibitors (SGLT2is) have demonstrated cardiovascular and kidney benefits in patients with CKD with and without type 2 diabetes in studies involving participants with an eGFR ≥30, ≥25, or ≥20 ml/min per 1.73 m^2^ (CREDENCE [Canagliflozin and Renal Events in Diabetes with Established Nephropathy Clinical Evaluation], DAPA-CKD [Dapagliflozin and Prevention of Adverse Outcomes in Chronic Kidney Disease], and EMPA-KIDNEY [the Study of Heart and Kidney Protection With Empagliflozin], respectively).^4–6^ A reduction in cardiovascular event risk was also observed with glucagon-like peptide-1 receptor agonists in type 2 diabetes.^7–9^ Of the participants studied, ≤4%, 14%, and approximately 35% had an eGFR <30 ml/min per 1.73 m^2^ in the glucagon-like peptide-1 receptor agonist and CREDENCE trials, DAPA-CKD trial, and EMPA-KIDNEY trial, respectively.^4–11^ Additional options are needed to reduce the risk of CKD progression and cardiovascular events in this understudied population.^12^

Finerenone, a selective, nonsteroidal mineralocorticoid receptor antagonist, blocks mineralocorticoid receptor overactivation.^13–16^ Finerenone, in addition to maximum tolerated renin-angiotensin system inhibition, was evaluated in two complementary phase 3 trials, including participants across the CKD spectrum with concomitant type 2 diabetes. The FIDELIO-DKD (Finerenone in Reducing Kidney Failure and Disease Progression in Diabetic Kidney Disease; NCT02540993) trial demonstrated that finerenone significantly reduced the risk of the primary kidney composite outcome in participants with mean eGFR of 44.3 ml/min per 1.73 m^2^ and median urine albumin-to-creatinine ratio (UACR) of 852 mg/g.^17^ In the FIGARO-DKD (Finerenone in Reducing Cardiovascular Mortality and Morbidity in Diabetic Kidney Disease; NCT02545049) trial, finerenone significantly reduced the primary cardiovascular composite outcome risk in participants with less-advanced CKD.^18^

FIDELITY (Finerenone in Chronic Kidney Disease and Type 2 Diabetes: Combined FIDELIO-DKD and FIGARO-DKD trial programme analysis) is a prespecified, individual participant data pooled analysis of the FIDELIO-DKD and FIGARO-DKD trials.^19^ This exploratory analysis of the FIDELITY stage 4 CKD participant subgroup aims to evaluate the efficacy and safety of finerenone in this population.

## Methods

### Study Design and Participants

The FIDELITY analysis combines data from FIDELIO-DKD and FIGARO-DKD (*N*>13,000), two phase 3, randomized, double-blind, placebo-controlled, multicenter clinical trials. Trial design and study protocol details have been published previously and are provided in Supplemental Methods.^17–19^ Eligible participants in FIDELITY were adults with type 2 diabetes and CKD (UACR ≥30 to <300 mg/g and eGFR ≥25 to ≤90 ml/min per 1.73 m^2^ or UACR ≥300 to ≤5000 mg/g and eGFR ≥25 ml/min per 1.73 m^2^) treated with the maximum tolerated labeled dose of a renin-angiotensin system inhibitor.^19^ Participants with nondiabetic kidney disease, a recent history of dialysis for acute kidney failure or a kidney transplant, uncontrolled hypertension, or symptomatic chronic heart failure with reduced ejection fraction were excluded (Supplemental Table 1).^19^

Participants were randomized 1:1 to receive oral finerenone or placebo; initial dosing of study drug, 10 or 20 mg once daily, was based on eGFR at the screening visit, with possible up- or down-titration between 10 and 20 mg once daily based on serum potassium and eGFR. In this FIDELITY subgroup analysis, participants were grouped by CKD stage at baseline, either stage 4 (eGFR <30 ml/min per 1.73 m^2^) or stage 1–3 (eGFR ≥30 ml/min per 1.73 m^2^).

### Procedures and Outcomes

The cardiovascular outcome in FIDELITY was a composite of time to cardiovascular death, nonfatal myocardial infarction, nonfatal stroke, or hospitalization for heart failure. The kidney outcome was a composite of time to kidney failure, a decrease in eGFR from baseline of ≥57% sustained over ≥4 weeks, or kidney disease death. Kidney failure was defined as ESKD (initiation of long-term dialysis [for ≥90 days] or kidney transplant) or sustained decrease in eGFR to <15 ml/min per 1.73 m^2^ sustained over ≥4 weeks. Other efficacy outcomes were the individual components of the cardiovascular and kidney composites and changes in eGFR and UACR over time. All outcomes were prospectively adjudicated by an independent clinical event committee blinded to treatment assignment. Safety outcomes included investigator-reported adverse events (AEs), change in systolic BP, and change in serum potassium over time.

### Statistical Analysis

Statistical analyses were performed as described previously for the FIDELITY overall population.^19^ Most statistical analyses were prespecified exploratory evaluations for participants with stage 4 CKD. Time-to-event treatment outcomes were expressed as hazard ratios (HRs) with corresponding confidence intervals (CIs) from a stratified Cox regression model. The stratified Cox proportional hazards model was fitted using the stratification factors study, region (North America, Latin America, Europe, Asia, and other), eGFR category at screening (25 to <45, 45 to <60, and ≥60 ml/min per 1.73 m^2^), albuminuria category at screening (moderately increased and severely increased), and history of cardiovascular disease (present or absent). The *P* value for interaction was based on a stratified Cox proportional hazards model, including treatment, subgroup, and treatment-by-subgroup interaction. Cumulative incidences based on Aalen–Johansen accounting for mortality as a competing risk and corresponding numbers needed to treat were calculated in 1-year intervals for the composite outcomes. The proportional hazards assumption was checked for each kidney outcome using a model including a treatment×log(time) interaction. If this assumption was violated for an outcome, indicating a possible change in treatment effect over time, the classical HR was not provided. Instead, to investigate the treatment effect at the beginning of treatment, the HR up to 2 years of treatment was calculated. In addition, finerenone versus placebo risk differences over time based on Aalen–Johansen estimates were provided to assess the effect of treatment over time.^20^

An on-treatment sensitivity analysis was performed for outcomes considering only events occurring ≤30 days after study drug cessation in the full analysis set (Supplemental Methods, Supplemental Table 2). Annualized changes in eGFR from baseline to permanent discontinuation or end-of-study visit (*i.e.*, total eGFR slope) and from month 4 to permanent discontinuation or end-of-study visit (*i.e.*, chronic eGFR slope) were evaluated by means of an analysis of covariance model, including baseline eGFR, treatment group, and stratification factors as covariates. Time courses for least squares (LS) mean changes in eGFR from baseline and LS mean UACR ratios to baseline were assessed using a mixed-model analysis. Mixed-model factors included treatment group, region, eGFR category at screening, type of albuminuria at screening, cardiovascular disease history, time, treatment×time, study, study×treatment, log-transformed baseline value nested within type of albuminuria at screening, and log-transformed baseline value×time as covariates.

## Results

The FIDELITY analysis included 13,026 participants, with a median follow-up of 3.0 years.^19^ In this subgroup analysis, 13,023 participants with baseline eGFR data were included; of these, 890 (7%) participants had stage 4 CKD at baseline. Demographics and baseline characteristics of participants with stage 4 CKD were balanced between treatment arms (Table 1). Participants with stage 4 CKD as a whole had a mean eGFR of 26.9 ml/min per 1.73 m^2^ and a median UACR of 720 mg/g (Supplemental Table 3). Larger proportions of participants with stage 4 CKD identified as Black, were women, were aged 75 years or older, had serum potassium >5.0 mmol/L, and had systolic BP ≥160 mm Hg compared with participants with stage 1–3 CKD. Median UACR was higher in participants with stage 4 CKD (720 mg/g) versus stage 1–3 CKD (503 mg/g). Baseline use of *β*-blockers, calcium channel blockers, statins, loop diuretics, and potassium-lowering agents was higher in participants with stage 4 CKD, whereas use of metformin and sodium-glucose cotransporter-2 inhibitor at baseline was lower (Supplemental Table 3).

**Table 1:** Baseline demographics and clinical characteristics in participants with stage 4 CKD in Finerenone in Chronic Kidney Disease and Type 2 Diabetes^a^

Characteristic	Finerenone (*n*=440)	Placebo (*n*=450)
Age, yr	67 (9)	67 (9)
**Sex, *n* (%)**		
Male	272 (62)	298 (66)
Female	168 (38)	152 (34)
**Race or ethnic group, *n* (%)**		
White	277 (63)	284 (63)
Black	31 (7)	33 (7)
Asian	105 (24)	98 (22)
Other^b^	27 (6)	35 (8)
Duration of diabetes, yr	17 (9)	18 (9)
HbA1c, %	7.6 (1.3)^c^	7.5 (1.3)
Systolic BP, mm Hg	136 (16)^d^	136 (16)
**Medical history at baseline, *n* (%)**		
History of cardiovascular disease	218 (50)	230 (51)
History of heart failure	37 (8)	50 (11)
**eGFR, ml/min per 1.73 m** ^**2**^	27 (2)	27 (2)
Minimum value	16	16
<25 ml/min per 1.73 m^2^, *n* (%)	81 (18)	81 (18)
UACR, mg/g, median (IQR)	741 (242–1556)	689 (243–1684)
**UACR, mg/g, *n* (%)**		
<30	9 (2)	7 (2)
30 to <300	123 (28)	116 (26)
≥300	308 (70)	327 (73)
Serum potassium, mmol/L	4.4 (0.5)	4.4 (0.5)
**Baseline medications, *n* (%)**		
Renin-angiotensin system inhibitors		
*ACEi*	150 (34)	139 (31)
*ARB*	290 (66)	309 (69)
Diuretics	276 (63)	312 (69)
Statins	347 (79)	338 (75)
Potassium-lowering agents^e^	12 (3)	23 (5)
Glucose-lowering medications		
*Insulin and analogs*	306 (70)	308 (68)
*GLP-1RA*	29 (7)	28 (6)
*SGLT2i*	5 (1)	8 (2)

Data are mean (SD) except where indicated. HbA1c, glycated hemoglobin; UACR, urine albumin-to-creatinine ratio; IQR, interquartile range; ACEi, angiotensin-converting enzyme inhibitor; ARB, angiotensin receptor blocker; GLP-1RA, glucagon-like peptide-1 receptor agonist; SGLT2i, sodium-glucose cotransporter-2 inhibitor.

a FIDELITY (combined Finerenone in Reducing Kidney Failure and Disease Progression in Diabetic Kidney Disease [FIDELIO-DKD] and Finerenone in Reducing Cardiovascular Mortality and Morbidity in Diabetic Kidney Disease [FIGARO-DKD] trial programme analysis) is a prespecified, individual participant data pooled analysis of the FIDELIO-DKD and FIGARO-DKD trials.

b Includes American Indian/native Alaskan, native Hawaiian/other Pacific Islander, not reported, and multiple.

c Data missing for *n*=2 participants.

d Data missing for *n*=1 participant.

e Including potassium binders.

**Figure 1:**
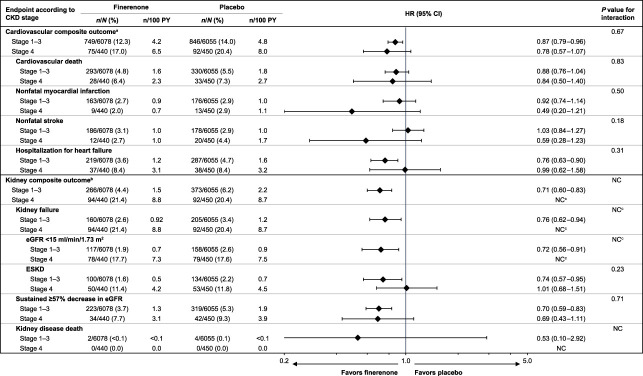
**Kidney and cardiovascular outcomes according to CKD stage at baseline.** ^a^Time to first onset of cardiovascular death, nonfatal myocardial infarction, nonfatal stroke, or hospitalization for heart failure. ^b^Time to first onset of kidney failure, sustained ≥57% decrease in eGFR from baseline over ≥4 weeks, or kidney disease death. ^c^Not calculated because the proportional hazards assumption was not met. CI, confidence interval; HR, hazard ratio; NC, not calculated; PY, person-years.

**Figure 2:**
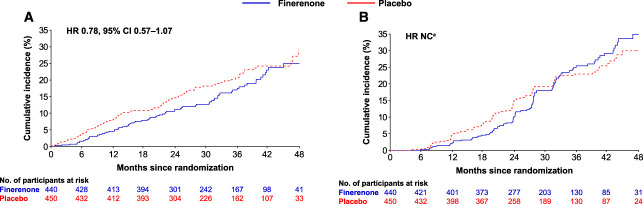
**Cardiovascular and kidney composite outcomes in participants with stage 4 CKD.** Aalen–Johansen estimates of (A) time to onset of the cardiovascular composite outcome in participants with stage 4 CKD and (B) time to onset of the kidney composite outcome in participants with stage 4 CKD. ^a^Not calculated because the proportional hazards assumption was not met.

Compared with participants with stage 1–3 CKD, a higher proportion of participants with stage 4 CKD experienced cardiovascular and kidney composite outcomes and their individual components apart from nonfatal myocardial infarction, nonfatal stroke, and kidney disease death (Figure 1).^20^ The cardiovascular composite outcome occurred in 75 of 440 (17%) and 92 of 450 (20%) participants with stage 4 CKD in the finerenone and placebo groups, respectively (HR, 0.78; 95% CI, 0.57 to 1.07). The effect of finerenone on the cardiovascular composite outcome was consistent between CKD subgroups (*P* value for interaction 0.67; Figure 1). Cumulative incidence analyses in participants with stage 4 CKD showed that the cardiovascular benefits of finerenone became apparent during the first year of the study (Figure 2A).^20^ There was no statistically significant evidence of heterogeneity in the effects of finerenone versus placebo on the components of the cardiovascular composite outcome, with *P* values for interaction ≥0.18 for every component (Figure 1). The estimated HRs of each component of the cardiovascular composite in participants with stage 4 CKD were similar or more favorable to finerenone than those with stage 1–3 CKD, with the exception of hospitalization for heart failure, for which a less favorable effect was observed in participants with stage 4 CKD (Figure 1).

**Figure 3:**
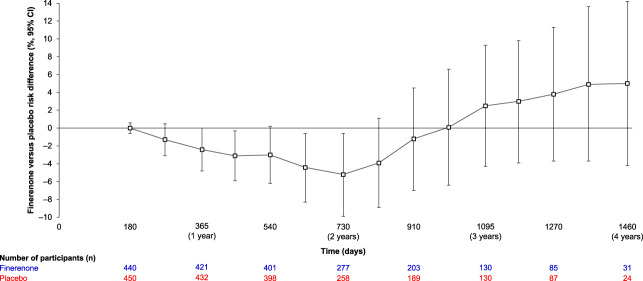
Finerenone versus placebo risk differences over time in participants with stage 4 CKD at baseline based on Aalen–Johansen estimates for time to onset of kidney failure, a sustained decrease of eGFR ≥57% from baseline over ≥4 weeks, or kidney disease death.

The kidney composite outcome occurred in 94 of 440 participants (21%) with stage 4 CKD treated with finerenone and 92 of 450 participants (20%) on placebo. In participants with stage 4 CKD, the proportional hazards assumption was not met according to the Cox proportional hazards model with treatment×log(time) interaction (*P* value for interaction <0.01). The HR at year 2 for the effect of finerenone versus placebo on the kidney composite outcome in these participants was 0.63 (95% CI, 0.42 to 0.95; *P* value 0.026). Cumulative incidence analyses in participants with stage 4 CKD showed a slower accumulation of first kidney events with finerenone versus placebo during the first 2 years of follow-up (between-group risk differences at 1 and 2 years: −2%; 95% CI, −5 to 0, and −5%; 95% CI, −10 to −1, respectively; Figure 2B, Table 2). However, subsequent events accumulated more rapidly in the finerenone group, with between-group differences of 3% (95% CI, −4 to 9) and 5% (95% CI, −4 to 14) at 3 and 4 years, respectively (Figure 3 and Table 2).

**Table 2:** Cumulative incidences based on Aalen–Johnson estimates for the cardiovascular and kidney composite outcomes in participants with stage 4 CKD

Time	Finerenone		Placebo		Risk Difference (95% CI)	NNT (95% CI)
	*n*/*N*	Cumulative Incidence (95% CI)	*n*/*N*	Cumulative Incidence (95% CI)		
**Cardiovascular composite outcome**						
1 yr	20/413	4.6 (2.9 to 6.8)	36/412	8.0 (5.7 to 10.7)	−3 (−7 to −0.2)	29 (15 to 434)
2 yr	47/301	11.2 (8.4 to 14.4)	64/304	14.7 (11.5 to 18.2)	−4 (−8 to 1)	29
3 yr	64/167	17.5 (13.6 to 21.7)	82/162	20.7 (16.7 to 24.9)	−3 (−9 to 3)	31
4 yr	75/41	25.0 (19.4 to 30.9)	92/33	29.1 (22.3 to 36.2)	−4 (−13 to 5)	24
**Kidney composite outcome**						
1 yr	9/401	2.1 (1.1 to 3.9)	20/398	4.5 (2.9 to 6.8)	−2 (−5 to 0)	41 (21 to 3349)
2 yr	39/277	10.1 (7.3 to 13.3)	62/258	15.2 (11.9 to 18.9)	−5 (−10 to −1)	19 (10 to 187)
3 yr	80/130	25.4 (20.6 to 30.6)	83/130	23.0 (18.6 to 27.6)	3 (−4 to 9)	−40^a^
4 yr	92/31	34.9 (28.2 to 41.8)	92/24	30.0 (24.0 to 36.2)	5 (−4 to 14)	−20^a^

*n* refers to the cumulative number of participants with events up to the day, inclusive. *N* refers to the number of participants at risk at the start of time point. CI, confidence interval; NNT, number needed to treat; NNH, number needed to harm.

a Negative NNT is equivalent to the NNH.

For the kidney failure and eGFR <15 ml/min per 1.73 m^2^ outcomes, the proportional hazards assumption was not met in participants with stage 4 CKD (*P* values for treatment×log[time] interaction <0.01 and 0.01, respectively). The HR at year 2 for time to kidney failure in participants with stage 4 CKD was 0.63 (95% CI, 0.42 to 0.95). All participants with stage 4 CKD who experienced the kidney composite outcome had a kidney failure event (Figure 1). The event history analysis revealed that the first kidney event for most participants was an eGFR of <15 ml/min per 1.73 m^2^ (Supplemental Figure 1), which most participants experienced (78 of 94 [83%] with finerenone and 79 of 92 [86%] with placebo). The HR at year 2 for time to eGFR <15 ml/min per 1.73 m^2^ in participants with stage 4 CKD was 0.54 (95% CI, 0.34 to 0.85; Figure 1). ESKD risk reduction was similar between CKD subgroups (*P* value for interaction 0.23; Figure 1). The HR for the risk of a sustained ≥57% decrease in eGFR was 0.69 (95% CI, 0.43 to 1.11) in participants with stage 4 CKD and 0.70 (95% CI, 0.59 to 0.83) in participants with stage 1–3 CKD, with a similar risk reduction between CKD subgroups (*P* value for interaction 0.71; Figure 1). No kidney disease deaths were observed in participants with stage 4 CKD; hence, a comparison between CKD subgroups could not be made.

**Figure 4:**
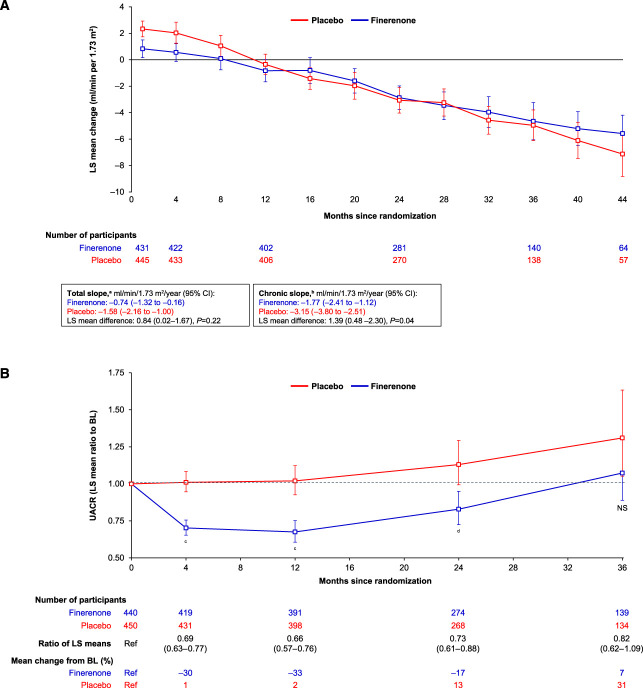
**Markers of kidney function over time in participants with stage 4 CKD.** (A) Mixed-model analysis of eGFR over time (LS mean change in eGFR from baseline by visit) in participants with stage 4 CKD at baseline. (B) Effect on albuminuria over time in participants with stage 4 CKD at baseline. ^a^Total slope assessed as annualized LS mean change in eGFR from baseline to the permanent discontinuation or end-of-study visit based on the ANCOVA model. ^b^Chronic slope assessed as annualized LS mean change in eGFR from month 4 to the permanent discontinuation or end-of-study visit based on ANCOVA model. ^c^*P* < 0.001. ^d^*P* = 0.0012. ANCOVA, analysis of covariance; BL, baseline; LS, least squares; NS, not significant; UACR, urine albumin-to-creatinine ratio.

Finerenone treatment significantly attenuated the annualized LS mean change in eGFR from month 4 to end of treatment (chronic eGFR slope) compared with placebo in participants with stage 4 CKD (Figure 4A). The chronic eGFR slope was −1.8 ml/min per 1.73 m^2^ per year in the finerenone group and −3.2 ml/min per 1.73 m^2^ per year in the placebo group (difference in LS means of 1.39 ml/min per 1.73 m^2^ [95% CI, 0.48 to 2.30; *P* = 0.04]). The annualized LS mean change in eGFR slope from baseline to end of treatment (total eGFR slope) was numerically lower in participants with stage 4 CKD who received finerenone (−0.7 ml/min per 1.73 m^2^ per year) versus those who received placebo (−1.6 ml/min per 1.73 m^2^ per year), resulting in a nonsignificant difference in LS means of 0.84 ml/min per 1.73 m^2^ (95% CI, 0.02 to 1.67; *P* = 0.22).

In participants with stage 4 CKD, the effect of finerenone on UACR between baseline and month 4 was consistent with that of the overall FIDELITY population (Supplemental Figure 2). Finerenone reduced UACR by 31% compared with placebo at month 4 in participants with stage 4 CKD (LS mean treatment ratio 0.69; 95% CI, 0.63 to 0.77; *P* < 0.001). A lower UACR in finerenone versus placebo recipients was maintained through to 24 months (Figure 4B).

AE incidences were generally balanced between treatment arms in participants with stage 4 and stage 1–3 CKD (Table 3). Hyperkalemia was the most common investigator-reported AE in participants with stage 4 CKD and was higher among participants receiving finerenone (26% for finerenone versus 13% for placebo; Table 3). However, the rate of hyperkalemia leading to permanent discontinuation was generally low in these participants (3% versus 2% for finerenone versus placebo, respectively; Table 3). Serum potassium levels increased after treatment with finerenone versus placebo (mean change from baseline at month 4: 0.26 [SD 0.51] versus 0.02 [SD 0.49], respectively) in participants with stage 4 CKD. However, the mean serum potassium level was stable over time thereafter (Supplemental Figure 3). Kidney-related AEs, including AKI, were generally balanced between treatment arms (Table 3). Participants with stage 4 CKD receiving finerenone had a modest reduction in mean systolic BP versus placebo (mean change from baseline at month 4: −2.0 mm Hg [SD 16.1] versus 1.7 mm Hg [SD 15.5], respectively; Supplemental Figure 4).

**Table 3:** Safety outcomes with finerenone and placebo in participants with stage 4 CKD

*n* (%)	Stage 4 CKD	
	Finerenone (*n*=438)	Placebo (*n*=448)
**Any treatment-emergent AE**	392 (90)	408 (91)
Related to study drug	118 (27)	84 (19)
Leading to treatment discontinuation	42 (10)	38 (9)
**Any treatment-emergent SAE**	149 (34)	188 (42)
Related to study drug	16 (4)	10 (2)
Leading to treatment discontinuation	19 (4)	15 (3)
**Kidney and urinary disorders**	86 (20)	105 (23)
AKI	24 (6)	33 (7)
**Worsening kidney function**		
Leading to discontinuation	7 (2)	9 (2)
Leading to hospitalization	17 (4)	18 (4)
**Hyperkalemia**	115 (26)	56 (13)
Leading to discontinuation	14 (3)	10 (2)
Leading to hospitalization	13 (3)	5 (1)

AE, adverse event; SAE, serious adverse event.

## Discussion

This exploratory subanalysis of the FIDELITY prespecified pooled dataset suggests an overall cardiovascular benefit and consistent safety profile of finerenone in a subgroup of 890 participants with type 2 diabetes and stage 4 CKD, with eGFR extending down to 25 ml/min per 1.73 m^2^ at screening. These data offer informative insights into the effect of finerenone in participants with stage 4 CKD and type 2 diabetes, a population that lacks treatment options and has previously been understudied.

Although a higher proportion of cardiovascular events occurred in participants with stage 4 CKD versus participants with stage 1–3 CKD, the overall effect of finerenone on the cardiovascular composite outcome and individual components was not significantly different across participants with varying CKD severity. Participants with stage 4 CKD have a higher risk of cardiovascular events.^21,22^ In this study, 17% and 21% of participants with stage 4 CKD experienced a cardiovascular and kidney event, respectively, highlighting the significant impact of cardiovascular events on these participants compared with participants with less-advanced CKD. Cumulative incidence analyses showed that the cardiovascular benefits of finerenone became apparent during the first 2 years of the study (Figure 2, Table 2).

Participants with stage 4 CKD are more likely to experience a higher level of kidney-related morbidity compared with those with less-advanced disease.^23^ Accordingly, higher incidence of the kidney composite outcome and its individual components were observed in these participants compared with those from the remaining participant population. A sensitivity analysis performed on the kidney outcomes data showed that because of an apparent interaction between the treatment effect and time, the Cox proportional hazards assumption (two-sided *P* value >0.05) was not met for the kidney composite outcome and the outcome components of kidney failure and eGFR <15 ml/min per 1.73 m^2^ in participants with stage 4 CKD. The use of these methods for stage 4 CKD participant data analysis limited the comparability of results obtained for this group with those of participants with stage 1–3 CKD (Figure 1).

Markedly, the cumulative incidence analysis in participants with stage 4 CKD suggested an initial delay in the time to experiencing a first kidney composite event (eGFR of <15 ml/min per 1.73 m^2^ for most participants) in the finerenone versus placebo recipients, supporting possible early kidney protection with finerenone (Figure 2, Supplemental Figure 1). For participants with stage 4 CKD, there was a slower accumulation of first kidney events in the finerenone versus placebo groups during the first 2 years of follow-up. Indeed, finerenone versus placebo risk differences over time suggested a reduction in the risk of kidney outcomes over the first 2 years. However, this effect was not consistent with the results observed beyond the 2-year time point (Figure 3, Table 2). Notably, risk difference CIs became larger and crossed 0, indicating a loss of precision after 2 years (Figure 3, Table 2).

The lack of observable reduction in the kidney composite beyond the 2-year time point in participants with stage 4 CKD may have been due to the severe state of irreversible kidney damage associated with this advanced disease, which might hinder long-term improvements.^24^ However, it could also be related to limited study power to assess such an effect or to limited ability of the predefined kidney composite outcome (which was selected to examine kidney disease progression in the whole study population) to reflect, in a similarly objective manner, kidney disease progression in participants at this advanced disease stage over the specific time frame. This notion is supported by the fact that in a similar manner to the overall FIDELITY population,^19^ in participants with stage 4 CKD, finerenone was associated with significant differences versus placebo in both intermediate kidney outcomes tested. A significant difference in the chronic eGFR slope was noted between finerenone and placebo, and the total eGFR slope was numerically lower in participants who received finerenone versus placebo. UACR was significantly reduced with finerenone by 31% at month 4 compared with baseline. Of note, a discrepancy between the effects of the active drug on the predefined composite kidney outcome versus the chronic eGFR slope was also noted in the EMPA-KIDNEY study, where empagliflozin did not seem to significantly affect the composite kidney outcome in participants with UACR <30 mg/g and UACR ≥30 to ≤300 mg/g but was associated with better preservation of the chronic eGFR slope versus placebo in both these participant subgroups.^6^

When considering the components of the kidney composite outcome, finerenone consistently reduced the risk of sustained ≥57% decrease in eGFR versus placebo in participants with stage 4 CKD. The proportion of participants experiencing a kidney failure event (eGFR <15 ml/min per 1.73 m^2^ or ESKD) was higher than those experiencing a sustained ≥57% decrease in eGFR in the stage 4 CKD subgroup. The opposite was true for the stage 1–3 CKD subgroup. This observation may be explained by the fact that a ≥57% decrease in eGFR from baseline in participants who began the trial with stage 4 CKD would invariably be experienced at an eGFR level below the <15 ml/min per 1.73 m^2^ threshold.

Comparing these results with recent trials of other treatment agents is difficult because of differences in entry criteria and efficacy outcomes. Nonetheless, a *post hoc* analysis of the CREDENCE trial of canagliflozin in CKD and type 2 diabetes, including 174 participants with eGFR <30 ml/min per 1.73 m^2^, showed that the kidney-protective effects of canagliflozin in these participants were consistent with those observed in participants with an eGFR ≥30 ml/min per 1.73 m^2^.^10^ In agreement with observations reported here, a 33% reduction in UACR was reported in the CREDENCE *post hoc* analysis with canagliflozin versus placebo, and the rate of eGFR decline was also reduced.^10^ Similar to FIDELITY, participants treated with dapagliflozin in the DAPA-CKD trial had an eGFR ≥25 ml/min per 1.73 m^2^, but type 2 diabetes was not a requirement for study entry.^5^ A subsequent analysis of 624 participants with stage 4 CKD (approximately 65% with type 2 diabetes) showed a consistent benefit of dapagliflozin in reducing the risk of major kidney and cardiovascular events, as well as attenuating progressive eGFR decline compared with participants with less-advanced CKD.^11^ In addition, in the EMPA-KIDNEY trial, empagliflozin was shown to reduce the risk of kidney disease progression over a median of 2 years of follow-up in a population including 2282 (35%) participants with eGFR <30 ml/min per 1.73 m^2^, with or without type 2 diabetes.

Safety findings in participants with stage 4 CKD were generally consistent with those observed in the overall FIDELITY population.^19^ Hyperkalemia incidence was higher in participants with stage 4 CKD who were treated with finerenone or placebo compared with participants with stage 1–3 CKD, consistent with a large-scale, real-world, observational study on hyperkalemia incidence by CKD stage and a *post hoc* safety analysis from FIDELIO-DKD, which showed that lower eGFR independently predicted hyperkalemia.^25,26^ However, there were few discontinuations (3% versus 2%) or hospitalizations (3% versus 1%) due to hyperkalemia in participants with stage 4 CKD in both the finerenone and placebo treatment arms, respectively; no deaths due to hyperkalemia were reported in this participant population, confirming the low absolute risk of clinically relevant events with finerenone (Table 3). Monitoring of serum potassium levels coupled with strategies to minimize the risk of hyperkalemia (*e.g.*, use of newer potassium-lowering agents) should be considered in participants with stage 4 CKD treated with finerenone.

Although FIDELITY was designed to include a large participant population with type 2 diabetes across a broad spectrum of CKD, participants with stage 4 CKD in this exploratory subanalysis accounted for <10% of the overall population, limiting the statistical power associated with these findings.

In summary, this exploratory subanalysis shows that the overall cardiovascular benefits and safety profile of finerenone were consistent across participants with stages 1–4 CKD. Further research is warranted because the effect of finerenone on the composite kidney outcome in participants with stage 4 CKD was inconsistent in early versus late years of follow-up, with a notable loss of precision over time. However, finerenone consistently showed improvements in markers of kidney injury (as shown by a reduction in UACR) and function (better preservation of eGFR in the chronic phase) versus placebo in participants with stage 4 CKD. These findings suggest that finerenone could provide cardiovascular benefits in participants with stage 4 CKD and type 2 diabetes, while also reducing albuminuria and the rate of eGFR decline.

## Supplementary Material

## Data Availability

Data from this study will be made available in the public domain. The electronic repository and date of data availability will be confirmed by Bayer AG.

## Supplemental Material

Supplemental Summary 1.

Supplemental Methods.

Supplemental Table 1. Pooled analysis study details.

Supplemental Table 2. Sensitivity analysis—cardiovascular and kidney composite outcomes in the on-treatment analysis according to the baseline CKD stage.

Supplemental Table 3. Baseline demographics and clinical characteristics in participants according to the baseline CKD stage.

Supplemental Figure 1. Event history for the kidney composite outcome in participants with stage 4 CKD.

Supplemental Figure 2. UACR levels at baseline and month 4 with finerenone and placebo according to the CKD stage at baseline.

Supplemental Figure 3. Mean change in serum potassium over time in participants with stage 4 CKD at baseline.

Supplemental Figure 4. Mean change in systolic BP over time in participants with stage 4 CKD at baseline.

## References

[1] Kidney Disease Improving Global Outcomes KDIGO Diabetes Work Group. KDIGO 2020 clinical practice guideline for diabetes management in chronic kidney disease. Kidney Int. 2020;98(4S):S1–S115. doi:10.1016/j.kint.2020.06.019 32998798

[2] American Diabetes Association Professional Practice Committee. Chronic kidney disease and risk management: standards of medical care in diabetes—2022. Diabetes Care. 2022;45(suppl 1):S175–S184. doi:10.2337/dc22-s011 34964873

[3] Gansevoort RT Correa-Rotter R Hemmelgarn BR Jafar TH Heerspink HJ Mann JF. Chronic kidney disease and cardiovascular risk: epidemiology, mechanisms, and prevention. Lancet. 2013;382(9889):339–352. doi:10.1016/s0140-6736(13)60595-4 23727170

[4] Perkovic V Jardine MJ Neal B Bompoint S Heerspink HJL Charytan DM. Canagliflozin and renal outcomes in type 2 diabetes and nephropathy. N Engl J Med. 2019;380(24):2295–2306. doi:10.1056/nejmoa1811744 30990260

[5] Heerspink HJL Stefánsson BV Correa-Rotter R Chertow GM Greene T Hou FF. Dapagliflozin in patients with chronic kidney disease. N Engl J Med. 2020;383(15):1436–1446. doi:10.1056/nejmoa2024816 32970396

[6] The EMPA-KIDNEY Collaborative Group, Herrington WG Staplin N Wanner C Empagliflozin in patients with chronic kidney disease. N Engl J Med. 2023;388(2):117–127. doi:10.1056/NEJMoa2204233 36331190 PMC7614055

[7] Marso SP Daniels GH Brown-Frandsen K Kristensen P Mann JFE Nauck MA. Liraglutide and cardiovascular outcomes in type 2 diabetes. N Engl J Med. 2016;375(4):311–322. doi:10.1056/nejmoa1603827 27295427 PMC4985288

[8] Gerstein HC Colhoun HM Dagenais GR Diaz R Lakshmanan M Pais P. Dulaglutide and cardiovascular outcomes in type 2 diabetes (REWIND): a double-blind, randomised placebo-controlled trial. Lancet. 2019;394(10193):121–130. doi:10.1016/S0140-6736(19)31149-3 31189511

[9] Marso SP Bain SC Consoli A Eliaschewitz FG Jodar E Leiter LA. Semaglutide and cardiovascular outcomes in patients with type 2 diabetes. N Engl J Med. 2016;375(19):1834–1844. doi:10.1056/nejmoa1607141 27633186

[10] Bakris G Oshima M Mahaffey KW Agarwal R Cannon CP Capuano G. Effects of canagliflozin in patients with baseline eGFR <30 ml/min per 1.73 m^2^: subgroup analysis of the randomized CREDENCE trial. Clin J Am Soc Nephrol. 2020;15(12):1705–1714. doi:10.2215/CJN.10140620 33214158 PMC7769025

[11] Chertow GM Vart P Jongs N Toto RD Gorriz JL Hou FF. Effects of dapagliflozin in stage 4 chronic kidney disease. J Am Soc Nephrol. 2021;32(9):2352–2361. doi:10.1681/ASN.2021020167 34272327 PMC8729835

[12] Szczech LA Stewart RC Su HL DeLoskey RJ Astor BC Fox CH. Primary care detection of chronic kidney disease in adults with type-2 diabetes: the ADD-CKD Study (awareness, detection and drug therapy in type 2 diabetes and chronic kidney disease). PLoS One. 2014;9(11):e110535. doi:10.1371/journal.pone.0110535 25427285 PMC4245114

[13] Georgianos PI Agarwal R. Mineralocorticoid receptor antagonism in chronic kidney disease. Kidney Int Rep. 2021;6(9):2281–2291. doi:10.1016/j.ekir.2021.05.027 34514191 PMC8418944

[14] Kawanami D Takashi Y Muta Y Oda N Nagata D Takahashi H. Mineralocorticoid receptor antagonists in diabetic kidney disease. Front Pharmacol. 2021;12:754239. doi:10.3389/fphar.2021.754239 34790127 PMC8591525

[15] Agarwal R Kolkhof P Bakris G Bauersachs J Haller H Wada T. Steroidal and non-steroidal mineralocorticoid receptor antagonists in cardiorenal medicine. Eur Heart J. 2021;42(2):152–161. doi:10.1093/eurheartj/ehaa736 33099609 PMC7813624

[16] Kintscher U Bakris GL Kolkhof P. Novel non-steroidal mineralocorticoid receptor antagonists in cardiorenal disease. Br J Pharmacol. 2022;179(13):3220–3234. doi:10.1111/bph.15747 34811750

[17] Bakris GL Agarwal R Anker SD Pitt B Ruilope LM Rossing P. Effect of finerenone on chronic kidney disease outcomes in type 2 diabetes. N Engl J Med. 2020;383(23):2219–2229. doi:10.1056/nejmoa2025845 33264825

[18] Pitt B Filippatos G Agarwal R Anker SD Bakris GL Rossing P. Cardiovascular events with finerenone in kidney disease and type 2 diabetes. N Engl J Med. 2021;385(24):2252–2263. doi:10.1056/nejmoa2110956 34449181

[19] Agarwal R Filippatos G Pitt B Anker SD Rossing P Joseph A. Cardiovascular and kidney outcomes with finerenone in patients with type 2 diabetes and chronic kidney disease: the FIDELITY pooled analysis. Eur Heart J. 2022;43(6):474–484. doi:10.1093/eurheartj/ehab777 35023547 PMC8830527

[20] Boyd AP Kittelson JM Gillen DL. Estimation of treatment effect under non-proportional hazards and conditionally independent censoring. Stat Med. 2012;31(28):3504–3515. doi:10.1002/sim.5440 22763957 PMC3876422

[21] Vallianou NG Mitesh S Gkogkou A Geladari E. Chronic kidney disease and cardiovascular disease: is there any relationship? Curr Cardiol Rev. 2018;15(1):55–63. doi:10.2174/1573403x14666180711124825 PMC6367692 29992892

[22] Jankowski J Floege J Fliser D Böhm M Marx N. Cardiovascular disease in chronic kidney disease: pathophysiological insights and therapeutic options. Circulation. 2021;143(11):1157–1172. doi:10.1161/circulationaha.120.050686 33720773 PMC7969169

[23] Sud M Tangri N Pintilie M Levey AS Naimark DM. Progression to stage 4 chronic kidney disease and death, acute kidney injury and hospitalization risk: a retrospective cohort study. Nephrol Dial Transplant. 2016;31(7):1122–1130. doi:10.1093/ndt/gfv389 26590389

[24] Gjorgjievski N Dzekova-Vidimliski P Gerasimovska V Pavleska-Kuzmanovska S Gjorgievska J Dejanov P. Primary failure of the arteriovenous fistula in patients with chronic kidney disease stage 4/5. Open Access Maced J Med Sci. 2019;7(11):1782–1787. doi:10.3889/oamjms.2019.541 31316658 PMC6614255

[25] Einhorn LM Zhan M Hsu VD Walker LD Moen MF Seliger SL. The frequency of hyperkalemia and its significance in chronic kidney disease. Arch Intern Med. 2009;169(12):1156–1162. doi:10.1001/archinternmed.2009.132 19546417 PMC3544306

[26] Agarwal R Joseph A Anker SD Filippatos G Rossing P Ruilope LM. Hyperkalemia risk with finerenone: results from the FIDELIO-DKD trial. J Am Soc Nephrol. 2022;33(1):225–237. doi:10.1681/ASN.2021070942 34732509 PMC8763180

