# Supplementary material for: Outcomes with Finerenone in Participants with Stage 4 CKD and Type 2 Diabetes: A FIDELITY Subgroup Analysis
Source: Clin J Am Soc Nephrol. 2023 Apr 7;18(5):602–12. doi: 10.2215/CJN.0000000000000149 (PMC10278789; doi:10.2215/CJN.0000000000000149)
Supplement: Supplementary file 1 [file cjasn-18-602-s001.pdf]

Supplemental Material

Table of Contents

Supplemental Material 1
Supplemental Methods 2
Randomization and Masking in the FIDELIO-DKD and FIGARO-DKD Trials 2
Prespecified On-Treatment Sensitivity Analysis 2
Supplemental Tables 3
Supplemental Table 1: Pooled Analysis Study Details[1] 3
Supplemental Table 2: Sensitivity Analysis – Cardiovascular and Kidney Composite Outcomes in the On-Treatment Analysis According to Baseline CKD Stage 5
Supplemental Table 3: Baseline Demographics and Clinical Characteristics in Participants According to Baseline CKD Stage 6
Supplemental Figures 9
Supplemental Figure 1: Event History for the Kidney Composite Outcome in Participants with Stage 4 CKD 9
Supplemental Figure 2: UACR Levels at Baseline and Month 4 with Finerenone and Placebo According to CKD Stage at Baseline 11
Supplemental Figure 3: Mean Change in Serum Potassium over Time in Participants with Stage 4 CKD at Baseline 12
Supplemental Figure 4: Mean Change in Systolic BP over Time in Participants with Stage 4 CKD at Baseline 13
Supplemental References 14

Supplemental Methods

Randomization and Masking in the FIDELIO-DKD and FIGARO-DKD Trials

Randomization was stratified by region (North America, Latin America, Europe, Asia, or other), albuminuria at screening (moderately increased and severely increased), eGFR at screening (25–<45, 45–<60, or ≥60 ml/min/1.73 m^2), and history of cardiovascular disease (present or absent). Baseline eGFR was based on the eGFR value from Visit 1 or the last nonmissing eGFR value. All participants and study personnel (except the independent data-monitoring committee) were masked to treatment allocation.

Prespecified On-Treatment Sensitivity Analysis

A prespecified on-treatment sensitivity analysis that included all events from randomization up to 30 days after last intake of study drug confirmed the results of the main analysis for the cardiovascular and kidney composite outcomes (Supplemental Table 4).

Supplemental Tables

Supplemental Table 1: Pooled Analysis Study Details[1]

**Study name**	FIDELIO-DKD[2]	FIGARO-DKD[3]
**Publication year**	2020	2021
**Study design**	Phase 3, randomized, double-blind, placebo-controlled, multicenter clinical trial	Phase 3, randomized, double-blind, placebo-controlled, multicenter clinical trial
**Sample size**[a]	5734	7437
**Inclusion criteria**	• Age ≥18 years<br>• Type 2 diabetes and CKD defined as UACR of 30–<300 mg/g, 25–<60 ml/min/1.73 m^2, and diabetic retinopathy, *or* UACR 300–5000 and eGFR 25–<75 ml/min/1.73 m^2<br>• Maximum tolerated dose of a RAS inhibitor<br>• Serum potassium ≤4.8 mmol/l	• Age ≥18 years<br>• Type 2 diabetes and CKD defined as UACR of 30–<300 mg/g and eGFR 25–90 ml/min/1.73 m^2, *or* UACR 300–5000 mg/g and eGFR ≥60 ml/min/1.73 m^2<br>• Maximum tolerated dose of a RAS inhibitor<br>• Serum potassium ≤4.8 mmol/l
**Exclusion criteria**	• Known significant nondiabetic kidney disease<br>• Uncontrolled hypertension[b]<br>• HbA_{1c} >12%<br>• Systolic BP <90 mm Hg at run-in visit<br>• Heart failure with reduced ejection fraction (NYHA II–IV) at run-in visit<br>• Recent cardiovascular event<br>• Dialysis for acute kidney failure<br>• Kidney transplant	• Known significant nondiabetic kidney disease<br>• Uncontrolled hypertension[b]<br>• HbA_{1c} >12%<br>• Systolic BP <90 mm Hg at run-in visit<br>• Heart failure with reduced ejection fraction (NYHA II–IV) at run-in visit<br>• Recent cardiovascular event<br>• Dialysis for acute kidney failure<br>• Kidney transplant
**Follow-up period, median**	2.6 years	3.4 years
**Primary outcome**	Time to kidney failure, a sustained decrease of at least 40% in eGFR from baseline, or kidney disease death	Time to cardiovascular death, nonfatal myocardial infarction, nonfatal stroke, or hospitalization for heart failure
**Secondary outcome**	Time to cardiovascular death, nonfatal myocardial infarction, nonfatal stroke, or hospitalization for heart failure	Time to kidney failure, a sustained decrease of at least 40% in eGFR from baseline, or kidney disease death
**Trial registry information**	NCT02540993	NCT02545049

The table shows the key features of the FIDELIO-DKD and FIGARO-DKD studies that comprised the FIDELITY prespecified pooled analysis, including publication year, study design, sample size, eligibility criteria, median follow-up, primary and main secondary endpoints, and links to the trials' ClinicalTrials.gov webpages.

BP, blood pressure; CKD, chronic kidney disease; eGFR, estimated glomerular filtration rate; FIDELIO-DKD, FInerenone in reducing kiDnEy faiLure and dIsease prOgression in Diabetic Kidney Disease; FIGARO-DKD, FInerenone in reducinG cArdiovascular moRtality and mOrbidity in Diabetic Kidney Disease; HbA_{1c}, glycated hemoglobin; NYHA, New York Heart Association; RAS, renin–angiotensin system; UACR, urine albumin-to-creatinine ratio.

[a]A total of 145 randomized participants (60 participants in FIDELIO-DKD and 85 participants in FIGARO-DKD) were prospectively excluded, prior to database lock, from all analyses because of critical Good Clinical Practice violations. This affected one site in the USA that was subsequently closed during the conduct of the trial, leading to the exclusion of 66 participants. In addition, during trial conduct it was detected that several participants were randomized simultaneously at multiple trial sites in the same locality in Florida, USA. This led to the prospective exclusion of a total of 79 participant IDs. [b]Mean sitting Systolic BP ≥170 mm Hg or mean sitting Diastolic BP ≥110 mm Hg at the run-in visit, or mean sitting Systolic BP ≥160 mm Hg or mean sitting Diastolic BP ≥100 mm Hg at the screening visit.

Supplemental Table 2: Sensitivity Analysis – Cardiovascular and Kidney Composite Outcomes in the On-Treatment Analysis According to Baseline CKD Stage

	Finerenone		Placebo		Hazard ratio (95% CI)	*P* value for interaction
	n (%)	Events per 100 PY	*n* (%)	Events per 100 PY		
Cardiovascular composite outcome						
Stage 1–3 CKD	572/6078 (9)	3.6	690/6055 (11)	4.37	0.82 (0.73–0.92)	0.61
Stage 4 CKD	48/440 (11)	5.19	67/450 (15)	7.08	0.69 (0.47–1.01)	
Kidney composite outcome						
Stage 1–3 CKD	163/6078 (3)	1.02	260/6055 (4.3)	1.62	0.63 (0.51–0.76)	0.05
Stage 4 CKD	61/440 (14)	6.56	65/450 (14.4)	6.83	0.96 (0.66–1.39)	

CI, confidence interval; CKD, chronic kidney disease; PY, person-years.

Supplemental Table 3: Baseline Demographics and Clinical Characteristics in Participants According to Baseline CKD Stage

**Characteristic**	**Stage 1–3 CKD (*n*=12,133)**	**Stage 4 CKD (*n*=890)**
Age, years	65 (10)	67 (9)
Age, years, *n* (%)		
<65	5574 (46)	313 (35)
65–74	4805 (40)	415 (47)
≥75	1754 (15)	162 (18)
Sex, *n* (%)		
Male	8518 (70)	570 (64)
Female	3615 (30)	320 (36)
Race or ethnic group, *n* (%)		
White	8307 (67)	561 (63)
Black/African American	458 (4)	64 (7)
Asian	2691 (22)	203 (23)
Other[a]	677 (6)	62 (7)
Duration of diabetes, years	15 (9)	18 (9)
HbA_{1c}, %	7.7 (1.4)[b]	7.6 (1.3)[c]
Systolic BP, mm Hg	137 (14)[d]	136 (16)[c]
Systolic BP, mm Hg, *n* (%)[e]		
<130	3658 (30)	291 (33)
130–<160	8022 (66)	547 (62)
≥160	452 (4)	50 (6)
Diastolic BP, mm Hg	77 (10)[d]	73 (11)[c]
hsCRP, mg/l	4.6 (9.2)	6.0 (16.1)
BMI, kg/m^2	31.3 (6.0)[f]	31.2 (6.5)[c]
Current smoker, *n* (%)	1996 (16.5)	97 (10.9)

Medical history at baseline, *n* (%)		
Hypertension	11,685 (96.3)	878 (98.7)
Diabetic retinopathy	4554 (37.5)	398 (44.7)
Diabetic neuropathy	3293 (27.1)	207 (23.3)
History of cardiovascular disease	5485 (45.2)	448 (50.3)
History of heart failure	919 (7.6)	87 (9.8)
eGFR, ml/min/1.73 m^2	59.8 (20.7)	26.9 (2.3)
UACR, mg/g, median (IQR)	503 (195–1114)	720 (242–1642)
UACR, mg/g, *n* (%)		
<30	214 (2)	16 (2)
30–<300	3860 (32)	239 (27)
≥300	8056 (66)	635 (71)
Serum potassium, mmol/l	4.3 (0.4)	4.4 (0.5)
Serum potassium, mmol/l, *n* (%)		
≤4.8	10,744 (89)	738 (83)
>4.8–≤5.0	742 (6)	65 (7)
>5.0	646 (5)	87 (10)
Baseline medications, *n* (%)		
Renin–angiotensin system inhibitors		
ACEi	4789 (40)	289 (33)
ARB	7336 (61)	599 (67)
Diuretics	6121 (50)	588 (66)
Loop diuretics	2406 (20)	396 (45)
Thiazide diuretics	2962 (24)	190 (21)
Statins	8712 (72)	685 (77)
Potassium-lowering agents[g]	147 (1)	35 (4)
β-blockers	5976 (49)	527 (59)

α-blocking agents	2504 (21)	269 (30)
Calcium channel blocker	6756 (56)	601 (68)
Platelet aggregation inhibitors[h]	6753 (56)	548 (62)
Glucose-lowering medications		
Insulin and analogues	7014 (58)	614 (69)
GLP-1RA	887 (7)	57 (6)
SGLT-2i	864 (7)	13 (2)
Metformin	7401 (61)	155 (17)
Sulfonylurea	3206 (26)	183 (21)
Alpha glucosidase inhibitor	614 (5)	42 (5)
DPP-4i	3008 (25)	270 (30)

Data are mean (SD) except where indicated.

ACEi, angiotensin-converting enzyme inhibitor; ARB, angiotensin receptor blocker; BMI, body mass index; BP, blood pressure; CKD, chronic kidney disease; DPP-4i, dipeptidyl peptidase-4 inhibitor; eGFR, estimated glomerular filtration rate; GLP-1RA, glucagon-like peptide-1 receptor agonist; HbA_{1c}, glycated hemoglobin; hsCRP, high-sensitivity C-reactive protein; IQR, interquartile range; SD, standard deviation; SGLT-2i; sodium-glucose co-transporter-2 inhibitor; UACR, urine albumin-to-creatinine ratio.

[a]Includes American Indian/native Alaskan, native Hawaiian/other Pacific Islander, not reported, and multiple. Data missing for the following participants: [b]*n*=17. [c]*n*=2. [d]*n*=1. [e]*n*=3. [f]*n*=31. [g]Including binders. [h]Excluding heparin.

Supplemental Figures

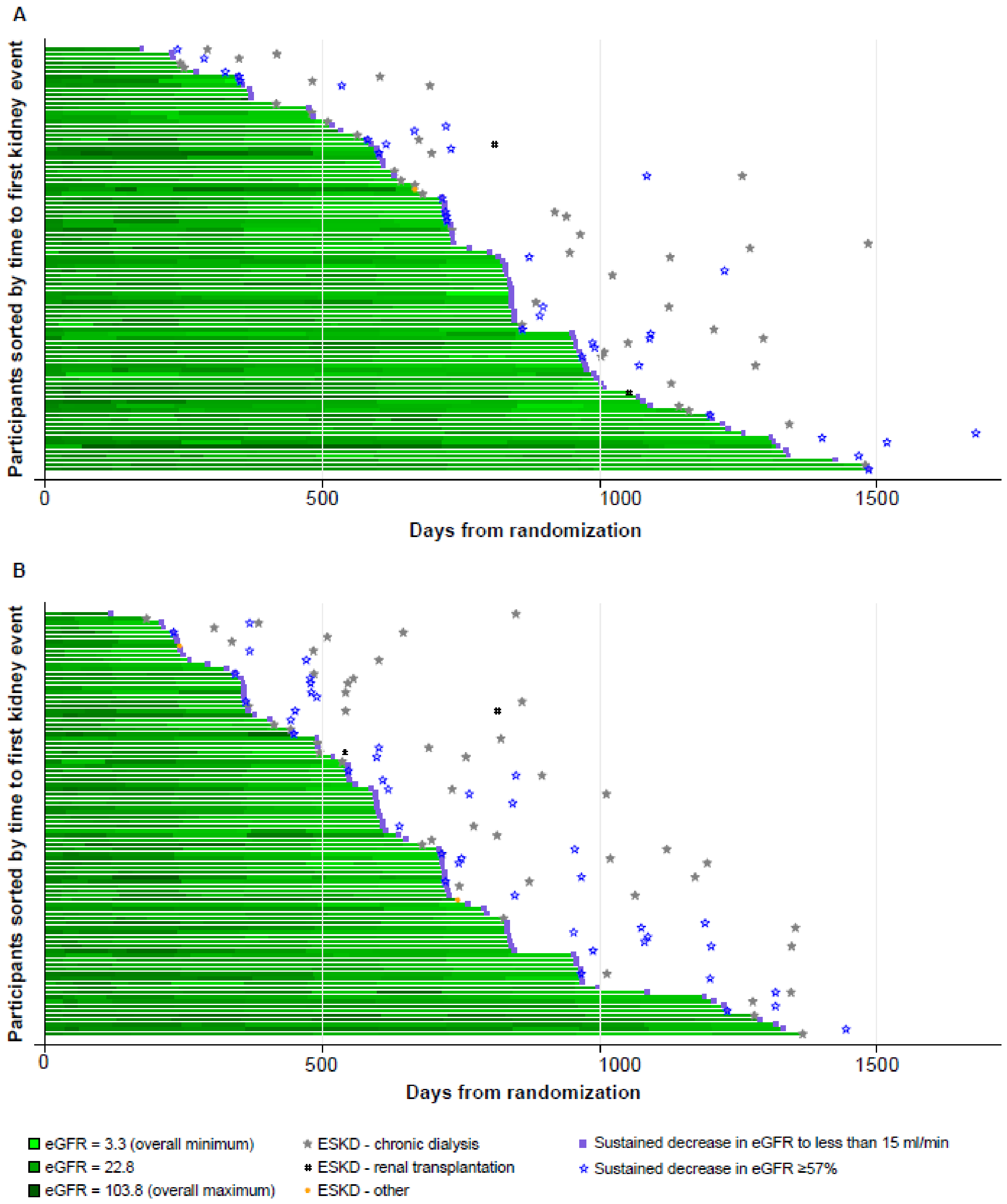

Supplemental Figure 1: Event History for the Kidney Composite Outcome in Participants with Stage 4 CKD

Each bar shows individual participant data from randomization until the day of first observed event in the kidney composite outcome, with the type of event specified according to the icons in the figure

legend, in participants s treated with (A) finerenone and (B) placebo. Bars are colored according to eGFR level until the first kidney event, and participants are ordered by time to first kidney event. CKD, chronic kidney disease; eGFR, estimated glomerular filtration rate; ESKD, end-stage kidney disease.

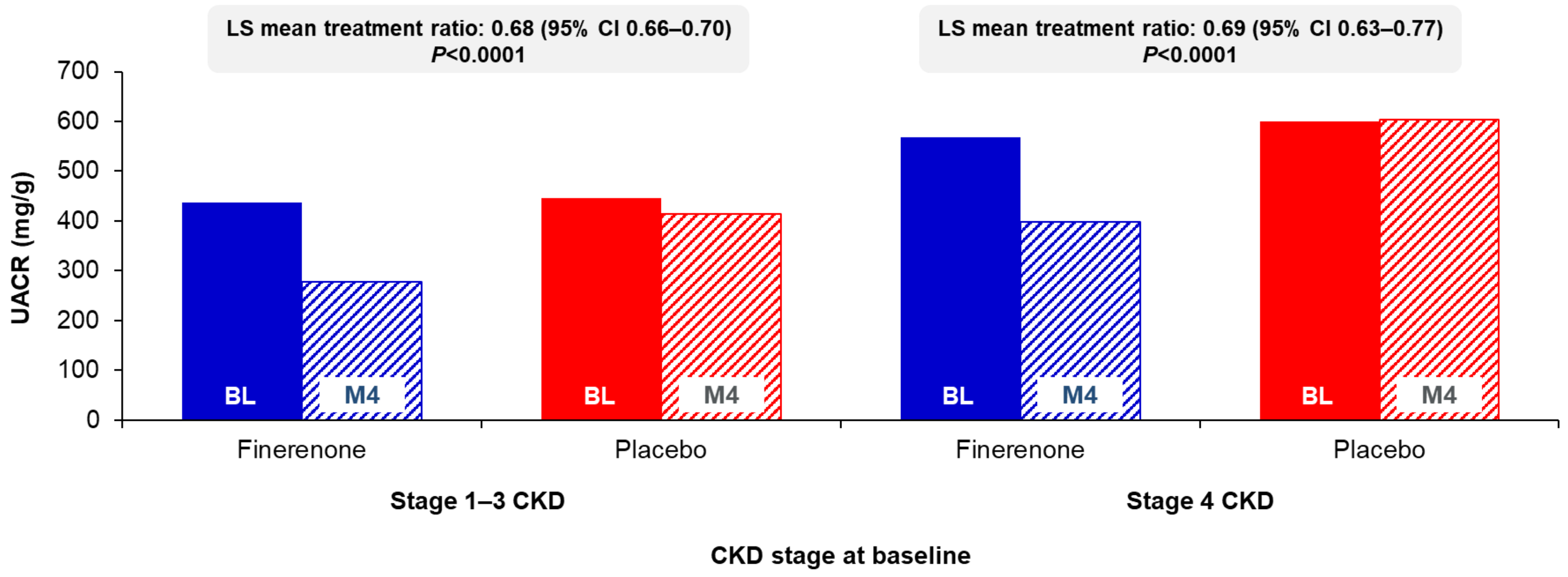

Supplemental Figure 2: UACR Levels at Baseline and Month 4 with Finerenone and Placebo According to CKD Stage at Baseline

BL, baseline; CI, confidence interval; CKD, chronic kidney disease; LS, least-squares; M, month; UACR, urine albumin-to-creatinine ratio.

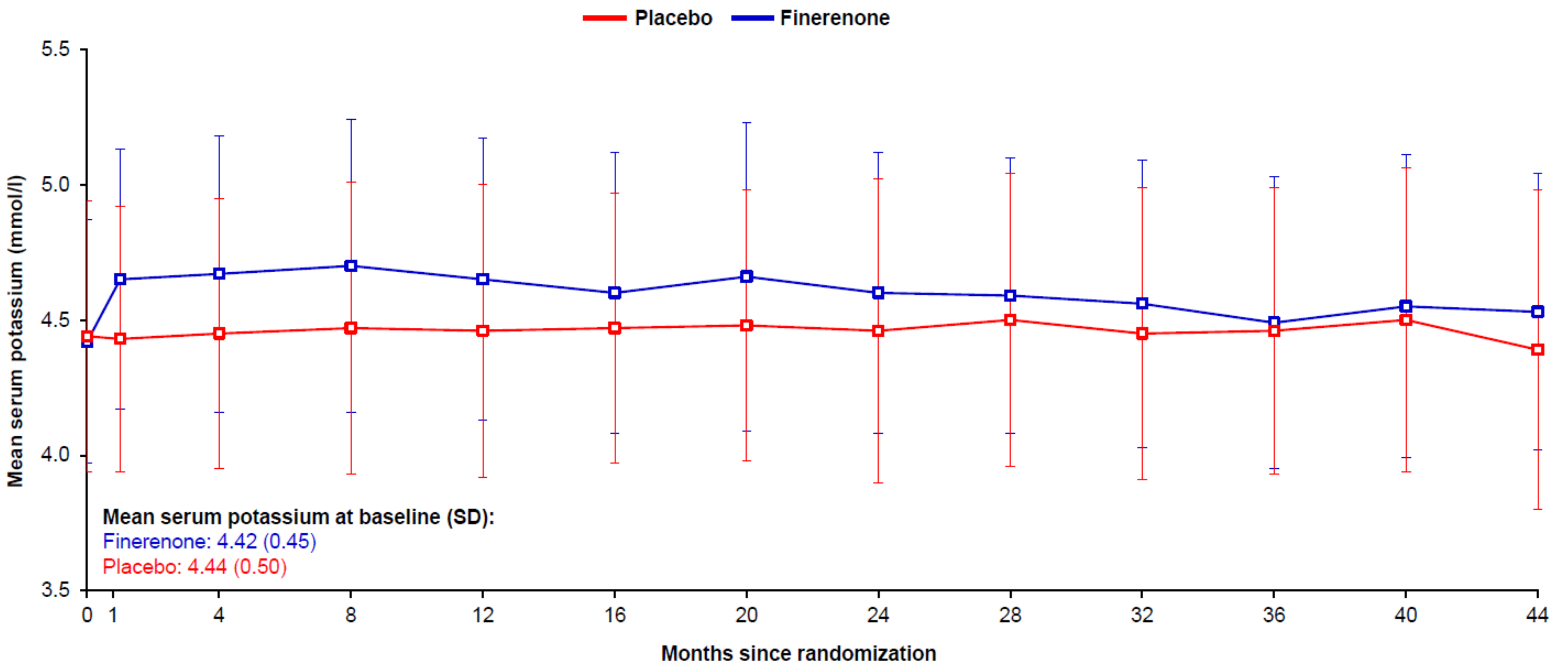

	0	4	8	12	16	20	24	28	32	36	40	44
**Number of participants (n)**												
Finerenone	438	414	401	402	380	335	282	237	187	151	115	70
Placebo	448	429	422	402	388	336	280	232	191	148	117	63
**Mean change from baseline (mmol/l)**												
Finerenone	Ref	0.26	0.28	0.23	0.18	0.24	0.18	0.16	0.12	0.05	0.09	0.07
Placebo	Ref	0.02	0.04	0.03	0.04	0.03	0.03	0.05	0.01	0.01	0.09	–0.11

Supplemental Figure 3: Mean Change in Serum Potassium over Time in Participants with Stage 4 CKD at Baseline

CKD, chronic kidney disease; SD, standard deviation.

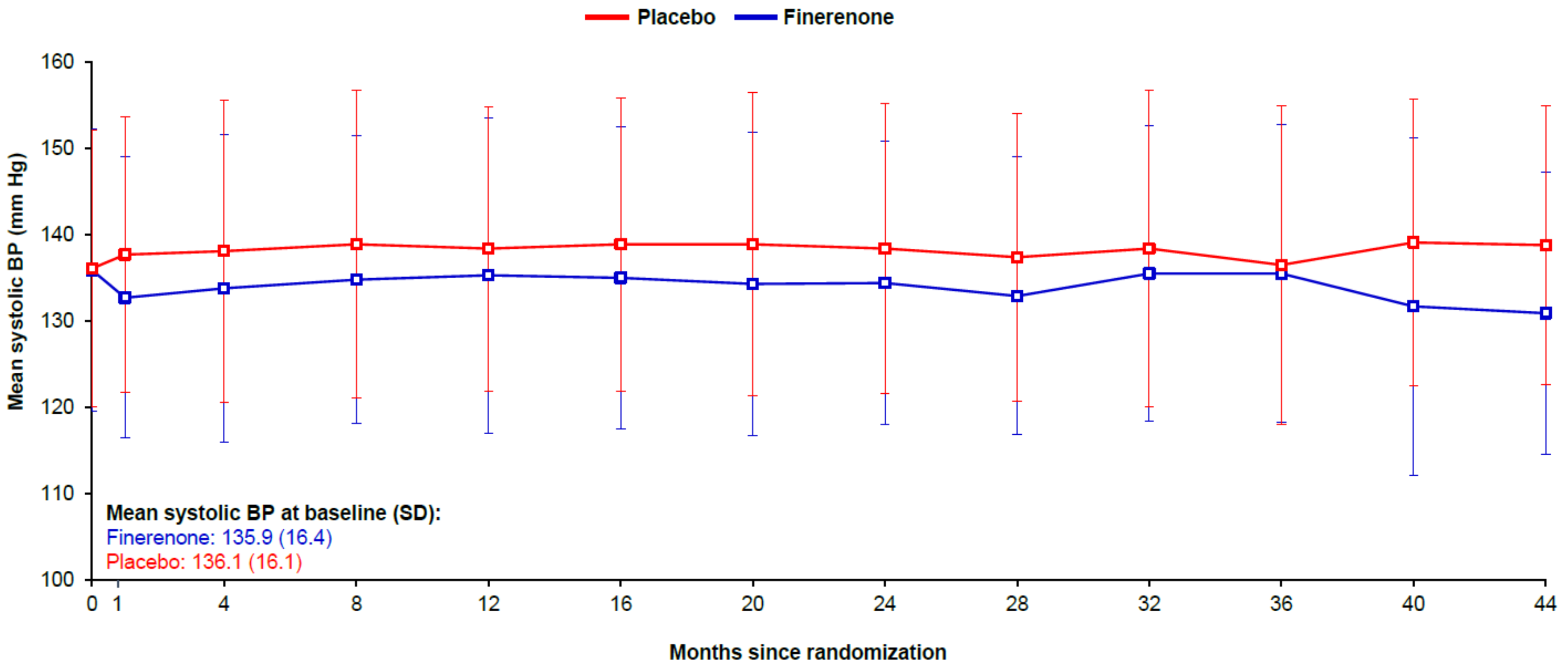

**Number of participants (n)**												
Finerenone	437	424	408	404	387	342	288	241	192	154	115	70
Placebo	448	436	428	407	390	345	291	237	193	151	119	64
**Mean change from baseline (mm Hg)**												
Finerenone	Ref	−2.0	−0.8	−0.6	−0.6	−0.7	−0.1	−1.2	1.5	2.0	−1.4	−2.1
Placebo	Ref	1.7	2.5	2.0	2.6	2.7	2.1	2.3	3.0	2.0	4.6	4.2

Supplemental Figure 4: Mean Change in Systolic BP over Time in Participants with Stage 4 CKD at Baseline

BP, blood pressure; CKD, chronic kidney disease; SD, standard deviation.

Supplemental References

1. Agarwal R, Filippatos G, Pitt B, Anker SD, Rossing P, Joseph A, et al.: Cardiovascular and kidney outcomes with finerenone in patients with type 2 diabetes and chronic kidney disease: the FIDELITY pooled analysis. Eur Heart J 43: 474–484, 2022
2. Bakris GL, Agarwal R, Anker SD, Pitt B, Ruilope LM, Rossing P, et al.: Effect of finerenone on chronic kidney disease outcomes in type 2 diabetes. N Engl J Med 383: 2219–2229, 2020
3. Pitt B, Filippatos G, Agarwal R, Anker SD, Bakris GL, Rossing P, et al.: Cardiovascular events with finerenone in kidney disease and type 2 diabetes. N Engl J Med 385: 2252–2263, 2021
